# Hydrops fetalis due to loss of function of hNav1.4 channel via compound heterozygous variants

**DOI:** 10.1038/s10038-024-01284-z

**Published:** 2024-08-21

**Authors:** Tomoya Kubota, Miho Nagata, Kazuko Takagi, Yasuki Ishihara, Kurumi Kojima, Yuka Uchikura, Reina Yamamoto, Ayumi Yonei, Erina Ozaki, Natsuki Kira, Satoe Takahashi, Kazuaki Homma, Yohei Miyashita, Minenori Eguchi-Ishimae, Norio Sakai, Yohihiro Asano, Yasushi Sakata, Keiichi Ozono, Mariko Eguchi, Masanori P. Takahashi

**Affiliations:** 1 Clinical Neurophysiology, Department of Clinical Laboratory and Biomedical Sciences, Osaka University Graduate School of Medicine, Suita, Osaka 5650871 Japan; 2 Department of Cardiovascular Medicine (IRUD Analysis Center), Osaka University Graduate School of Medicine, Suita, Osaka 5650871 Japan; 3 Department of Obstetrics and Gynecology, Ehime University Graduate School of Medicine, Touon, Ehime 7910295 Japan; 4 Division of Genetic Counselling, Osaka University Hospital, Suita, Osaka 5650871 Japan; 5 Division of Medical Genetics, Ehime University Hospital, Touon, Ehime 7910295 Japan; 6 Department of Otolaryngology-Head and Neck Surgery, Feinberg School of Medicine, Northwestern University, Chicago, IL 60611 USA; 7 The Hugh Knowles Center for Clinical and Basic Science in Hearing and Its Disorders, Northwestern University, Evanston, IL 60208 USA; 8 Department of Pediatrics, Ehime University Graduate School of Medicine, Touon, Ehime 7910295 Japan; 9 Department of Pediatrics, Osaka University Graduate School of Medicine, Suita, Osaka 5650871 Japan

**Keywords:** Neuromuscular disease, Reproductive disorders

## Abstract

Hydrops fetalis, characterized by abnormal fluid accumulation in fetuses, presents a significant risk of stillbirth and neonatal mortality. Although the etiology of nonimmune hydrops fetalis (NIHF) is multifaceted, recent studies have highlighted genetic factors as crucial determinants. This study focused on a family with three consecutive stillbirths, each with pronounced hydrops fetalis. Using whole-exome sequencing (WES), we identified compound heterozygous variants of the *SCN4A* gene encoding the voltage-gated sodium channel of the skeletal muscle (hNav1.4), c.2429T>A p.L810Q and c.4556T>C p.F1519S, in all three deceased infants. A functional analysis conducted using the whole-cell patch-clamp technique revealed loss-of-function defects in both variant channels, with F1519S exhibiting a complete loss of ionic current and L810Q showing a reduced channel opening. These findings support the pathogenicity of *SCN4A* variants in NIHF and underscore the significance of functional studies in elucidating genotype-phenotype correlations. Furthermore, our study emphasizes the diagnostic value of WES in cases of NIHF in where standard genetic testing fails to identify causative variants.

## Introduction

Hydrops fetalis, a severe fetal condition, is diagnosed when at least two fetal fluid collections, such as ascites, pleural effusion, pericardial effusion, and skin edema, are present [1, 2]. Non-immune hydrops fetalis (NIHF) affects approximately one in 1700–3000 pregnancies, posing a high risk of stillbirth and neonatal death. In infants with NIHF, neonatal mortality remains between 60% and 90%. The underlying mechanisms of NIHF encompassing cardiovascular disorders, lymphatic dysplasia, infection, chromosomal imbalance, twin-to-twin transfusion, placental abnormalities, and idiopathic disorders, without any underlying disorders. In a recent series involving 127 unresolved cases of NIHF, exome sequencing uncovered underlying genetic causes approximately one-third of cases [3].

Voltage-dependent sodium channels (Nav) play a crucial role in generating action potentials in excitable cells, such as neurons, cardiomyocytes, and skeletal muscle cells. Variants of the *SCN4A* gene encoding human Nav1.4 (hNav1.4), expressed in the skeletal muscle, are associated with autosomal-dominantly inherited muscle diseases such as sodium channel myotonia, paramyotonia congenita, hyperkalemic periodic paralysis, and hypokalemic periodic paralysis [4, 5]. In general, the underlying mechanism of these autosomal-dominant diseases is a gain of function defect of the channel, either stabilized opening in case of sodium channel myotonia, paramyotonia congenita, and hyperkalemic periodic paralysis, or creation of anomalous pore (gating pore) in case of hypokalemic periodic paralysis. Although most of these diseases are not life-threatening, they are known to be associated with a low quality of life [6, 7]. However, certain variants of the *SCN4A* gene have been implicated in life-threatening conditions, such as sudden infant death syndrome [8] and congenital myasthenia syndrome [9]. More recently, homozygous or compound heterozygous variants of *SCN4A* have been reported as causes of congenital myopathy, fetal hypokinesia, and fetal death, with autosomal recessive inheritance [10–18]. Functional analyses of some of these variants have revealed a loss of function defect of the channel as an underlying mechanism.

In this study, using whole-exome sequencing (WES), we identified compound heterozygous variants of the *SCN4A* gene within a family with three recurrent fetal or neonatal deaths associated with fetal hydrops. Functional analysis of the mutant channels revealed a loss-of-function defect, with one channel exhibiting no conduction and the other showing a reduced channel opening. Our case supports the hypothesis that *SCN4A* variants can lead to fetal death with hydrops and highlights the broadening spectrum of *SCN4A* channelopathy.

## Materials and Methods

### Genetic analysis

Following the approval of the study protocol by the Institutional Review Committees of Osaka University (Osaka, Japan) and Ehime University (Ehime, Japan), we secured written informed consent from the parents, explicitly granting permission for genetic analysis and publication in academic journals and meetings. Genomic DNA was extracted from the blood leukocytes of the parents and fragments of the umbilical cords from their three babies. Subsequently, WES analyses were performed using the SureSelect Human All Exon V6 Kit (Agilent Technology, Santa Clara, CA, USA) for exon capture and the NovaSeq6000 platform (Illumina, San Diego, CA, USA) with 100-bp paired-end reads for sequencing. FASTQ files were assessed for quality using FASTQC, and any low-quality reads were eliminated through trimmomatic-0.36. Quality-checked reads were aligned to GRCh37 using Burrows-Wheeler Aligner (http://bio-bwa.sourceforge.net/). Variants were called using the HaplotypeCaller module of the Genome Analysis Toolkit (GATK v.4.1.0), and annotated using ANNOVAR (http://annovar.openbioinformatics.org/en/latest/). The pathogenicity of the variants was scored using Combined Annotation-Dependent Depletion (CADD; http://cadd.gs.washington.edu/), Sorting Intolerant From Tolerant (SIFT, https://sift.bii.a-star.edu.sg/), and Protein Variation Effect Analyzer (PROVEAN; http://provean.jcvi.org/seq_submit.php). The variants were filtered by assessing the scores, genotypes, and minor allele frequency to identify the disease-causal variants. The minor allele frequency cutoff values were established based on the mode of inheritance as follows: autosomal dominant, 0.03; autosomal recessive, 0.05; de novo, 0.03; X-linked, 0.05; and compound heterozygous, 0.05.

### Molecular biology and generation of stable cell lines

Stable cell lines expressing wild-type hNav1.4 and mutant channels were generated, as previously described [19]. Briefly, wild-type human *SCN4A*, tagged with mTurquise2 (mTq2) at the C-terminus, followed by the ribosomal-skipping P2A sequence, and *SCN1B* (*SCN4A-mTq2-P2A-SCN1B*), was subcloned into an expression vector, *pSBtet-Pur* (Addgene, Watertown, MA, USA; cat.# 60507), using the NEBuilder HiFi DNA Assembly Cloning Kit (New England Biolabs, Ipswich, MA, USA; cat.# E5520). Missense variants, L810Q and F1519S, were introduced into *SCN4A*-expressing cells using a KOD-Plus Mutagenesis Kit (TOYOBO, Osaka, Japan). Stable HEK293T cell lines expressing hNav1.4 or its mutants in a doxycycline-inducible manner were generated through the transfection of both the aforementioned *pSBtet-Pur-SCN4A-mTq2-P2A-SCN1B* with/without a variant and *pCMV(CAT)T7-SB100* encoding SB100X transposase using the ViaFect™ Transfection Reagent (Promega, Madison, WI, USA; cat.# E4981) [20]. The transfected cells were selected in a medium containing 1 µg/mL of puromycin.

### Electrophysiology

The expression of hNav1.4 and its variants was induced by 2 µg/mL of doxycycline directly added to the culture media 3 h before recordings. Subsequently, the cells were placed on 12 mm glass coverslips for patch-clamp recordings. Ionic currents were recorded from the HEK293T cells using the whole-cell patch-clamp technique, employing Axopatch 200 B (Molecular Devices, San Jose, CA, USA). Data acquisition and analysis were performed using the Digidata 1550B digitizer (Molecular Devices) and the pCLAMP 11.1 software (Molecular Devices). Heat-polished glass microelectrodes with a resistance of 1.5 MΩ to 2.5 MΩ in external solution were utilized in the experiment. The electrode solution consisted of 105 mM CsF, 35 mM NaCl, 10 mM EGTA, and 10 mM HEPES (pH 7.4), while the bath solution comprised 140 mM NaCl, 4 mM KCl, 2 mM CaCl_2_, 1 mM MgCl_2_, 5 mM glucose, and 10 mM HEPES (pH 7.4). Recordings were performed at room temperature (23–25 °C). After achieving the whole-cell configuration, the membrane potential was held at −120 mV for 5 min to recover from slow-inactivated state. The currents were measured using activation and steady-state fast inactivation protocols, as shown in the figure insets. Cells exhibiting a peak current greater than 10 nA or less than 1 nA were excluded from the analysis. The kinetics of fast inactivation, ranging from −25 mV to +30 mV, were determined through a single exponential fit on ionic current decay. The channel conductance was calculated as previously described [21].

### Cell membrane-targeting assay

We evaluated the cell membrane-targeting efficacy of hNav1.4 and the variant channels as previously reported [19]. Briefly, cells were seeded on a 6-well plate, and the expression of mTq2-tagged hNav1.4 constructs was induced by 3 µg/mL doxycycline for 1 d. After washing once with PBS, the cells were incubated in 2 mL/well of 100 µM Sulfo-Cyanine3 NHS ester (Lumiprobe, Moscow, Russia; cat.# 21320) dissolved in ice-cold PBS for 30 min at 4 °C. The reaction was stopped by adding 200 µL of 100 mM glycine. Cells were collected and lysed on ice in 500 µL of lysis buffer (150 mM NaCl, 20 mM HEPES, pH 7.5, 1 mM EDTA, 20 mM DDM, 1 mM DTT, and 50 µg/mL leupeptin). The lysate was centrifuged at 16,000 × *g* for 5 min at 4 °C. Subsequently, 5 µL of GFP selector slurry (NanoTag Biotechnologies, Göttingen, Germany) was added to the supernatant and incubated for 30 min at 4 °C with end-over-end mixing using a rotator. The proteins bound with the GFP selector were collected by brief centrifugation and observed under a fluorescence microscope (Olympus, Tokyo, Japan). Merged images of the GFP selectors in the cyan and red channels were analyzed using FIJI [22] to determine the fluorescence signal intensities of mTq2 and Cy3.

### Statistical analyses

Statistical analyses were performed using the software programs Origin (OriginLab, Northampton, MA, USA) and JMP (JMP Statistical Discovery, Cary, NC, USA). Intergroup comparisons were performed using the Student’s t-test. Data are presented as mean ± standard error of the mean (S.E.M.)

### Cases

**Fig. 1:**
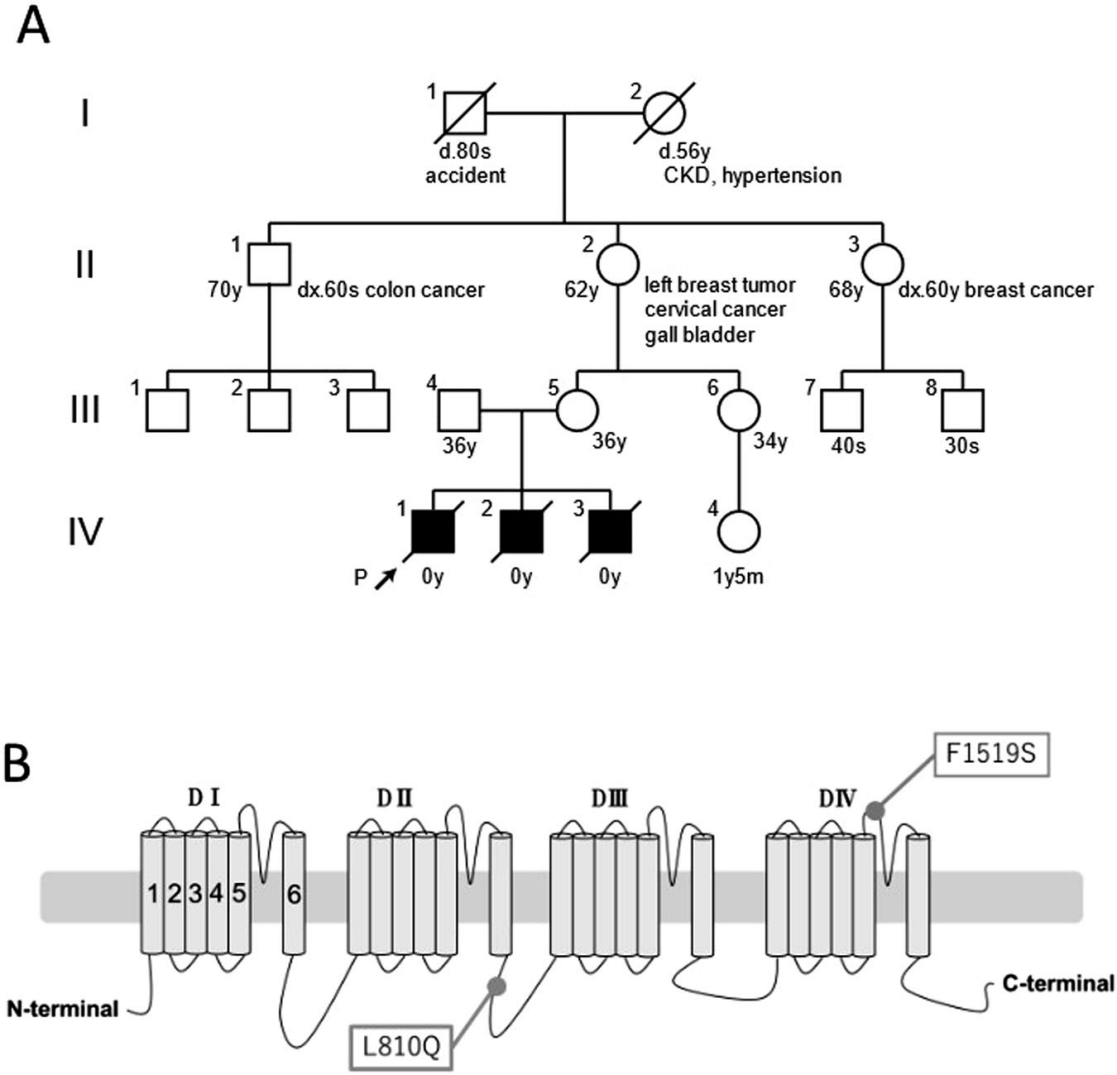
Genetic analysis of the family. **A** Family pedigree. The proband is indicated with the arrow. CKD chronic kidney disease. **B** Schematic representation of hNav1.4. Positions of identified variants, L810Q and F1519S, in this family are indicated

A nonconsanguineous 36-year-old woman (III-4) with no previous medical history experienced three consecutive stillbirths (Fig. 1A).

#### *Case* IV-1

The first pregnancy was conceived spontaneously and initially managed at a local clinic. However, she was later referred to a perinatal center because of the diagnosis of hydrops fetalis at 27 weeks of gestation. Ultrasonography revealed pleural effusion, ascites, and severe subcutaneous edema, confirming the diagnosis of severe hydrops fetalis. There was no evidence of infection or immune disease. Fetal MRI revealed lung hypoplasia associated with pleural effusion and ascites. The male baby was stillborn at 29 weeks of gestation with a body weight of 1796 g. No external abnormalities were noted except for generalized edema.

#### *Case* IV-2

The second pregnancy was conceived spontaneously at 25 years of age and was monitored at a nearby clinic from 9 weeks of gestation. Ultrasonography at that stage revealed a crown-rump length of 1.8 cm. At 24 weeks of gestation, the mother was referred to a perinatal center because of polyhydramnios. Subsequent ultrasonography revealed pleural effusion and thick subcutaneous edema of the head. Blood tests, including virological examination, showed no findings specific to any disease. Amniocentesis showed a normal male karyotype (46,XY). She complained of regular uterine contractions and delivered a male baby weighing 1870 g by vaginal delivery at 31 weeks’ gestation. The neonatal cardiac arrest was confirmed after the delivery, and no apparent malformation was found.

#### *Case* IV-3

The third fetus was conceived spontaneously. The mother was managed at a perinatal center from the beginning of the study. Ultrasound examination revealed subcutaneous edema of the fetal head at 20 weeks of gestation, followed by pleural effusion, subcutaneous edema, finger contracture, gastric bubble disappearance, and polyhydramnios at 24 weeks of gestation. Blood tests, including virological examinations, showed no specific findings. Amniocentesis revealed a normal male karyotype (46,XY). The fetal edema gradually worsened, leading to intrauterine fetal death confirmed at 28 weeks of gestation. Delivery was induced at 29 weeks of gestation, resulting in the stillbirth of a male fetus with a birth weight of 1500 g. Autopsy findings revealed no abnormalities other than generalized edema.

## Results

The whole-exome sequencing analysis in all three babies revealed two heterozygous missense variants of the *SCN4A* gene (NM_000334.4), c.2429T>A p.L810Q, also identified in the mother (III-4), and c.4556T>C p. F1519S, also identified in the father (III-5). Each variant was found in a heterozygous manner in the respective parents, indicating compound heterozygosity in the babies. Both variants were not registered in ClinVar and were rare variants with no available frequency information in gnomAD (https://gnomad.broadinstitute.org/) or jMorp (https://jmorp.megabank.tohoku.ac.jp/). In silico analysis suggested pathogenicity for both variants, with CADD, SIFT, and PROVEAN scores of 24.3, 0, and −7.4 for F1519S and 26.2, 0, and −5.78 for L810Q, respectively. According to the American College of Medical Genetics and Genomics guidelines, although both variants were categorized as variants of uncertain significance, specific criteria PM1, PM2, and PP3 were considered for F1519S and whereas only PM2 and PP3 were applied for L810Q.

**Fig. 2:**
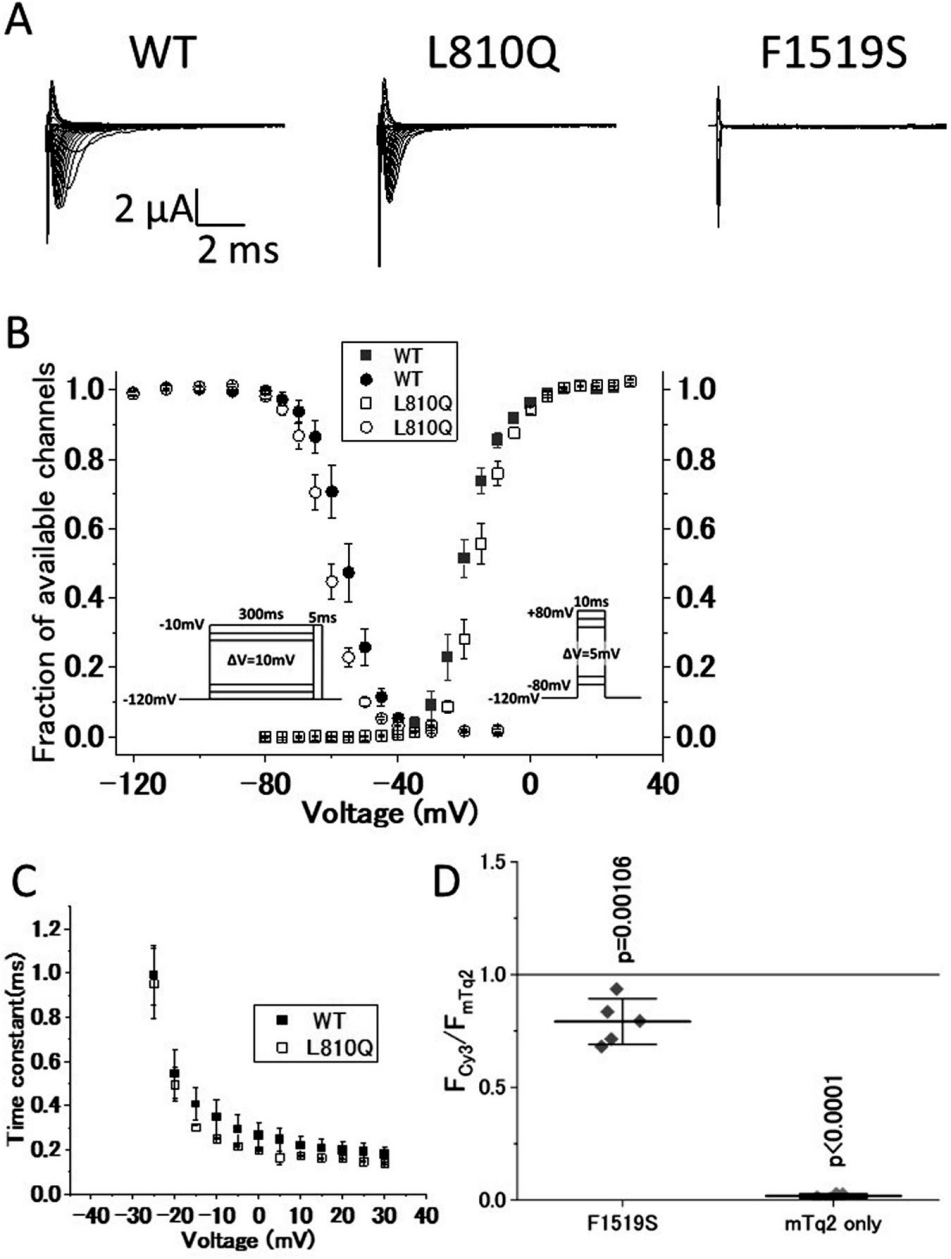
Functional comparison of *hNav1.4* with the variants. **A** Representative current traces for wild-type *hNav1.4* (WT), *hNav1.4* with *L810Q* (L810Q), and *hNav1.4* with *F1519S* (F1519S), expressed in HEK293T cells elicited by the activation protocol shown in Fig. 2B, right. **B** Voltage dependence of steady-state fast inactivation (circles) and activation (squares). Symbols are as follows: WT (filled) and L810Q (opened). **C** Voltage-dependent kinetics for fast inactivation ranging from −25 mV to +30 mV are shown. These data were obtained from single exponential decay of ionic current decay elicited by an activation protocol (shown in Fig. 2B, right). Error bars indicate SEM. **D** Cell membrane-targeting assay for WT, F1519S, and mTq2 alone. Note that mTq2 alone is the negative control. The Y-axis shows normalized values of the membrane-targeting efficacy of each clone (F1519S and mTq2 alone) with respect to WT (WT = 1.0, a gray line). F1519S exhibits similar or slightly lower membrane-targeting efficacy, but not zero, compared to WT, indicating that the F1519S channel significantly expresses in the plasma membrane, although it cannot conduct ionic currents as shown in Fig. 2A. Horizontal bars indicate mean ± SD

To elucidate the pathogenicity of these variants, we conducted functional analyses using the whole-cell patch-clamp technique. Three types of cell lines were prepared to express wild-type hNav1.4 (WT), hNav1.4 with L810Q, and hNav1.4 with F1519S. Representative ionic traces are shown in Fig. 2A. F1519S exhibited no ionic currents. The voltage dependence of activation and steady-state fast inactivation in L810Q are shown in Fig. 2B, and their parameters are listed in Table 1. The voltage dependence of activation in L810Q shifted towards depolarization by approximately 5 mV, making it difficult to activate the channel. Additionally, the voltage dependence of the steady-state fast inactivation shifted towards hyperpolarization by approximately 5 mV, indicating easy channel inactivation. Indeed, the fast inactivation kinetics of L810Q, ranging from −25 mV to +30 mV, were faster than those of the WT cells, as shown in Fig. 2C. These findings were consistent with the reduced opening of the L810Q channel compared to the WT, indicating loss-of-function defects in *hNav1.4* with the L810Q variant.

**Table 1:** Parameters of functional analysis

	Activation			Fast inactivation		
	*n*	V_1/2_ (mV)	k (mV/e-fold)	*n*	V_1/2_ (mV)	*k* (mV/e-fold)
WT	5	−20.22 ± 1.20	4.25 ± 0.35	5	−55.64 ± 1.59	4.82 ± 0.23
L810Q	7	−15.75 ± 1.09 *	4.09 ± 0.16	7	−60.95 ± 1.06 *	4.64 ± 0.33

Values indicate means ± SEM, standard error of mean. Asterisks indicate statistical significance, *p* < 0.05

Owing to the absence of observable ionic currents from F1519S, we investigated the expression of hNav1.4 with F1519S in the plasma membrane using a Cy3-based cell membrane-targeting assay, as previously reported. As shown in Fig. 2D, the expression efficacy of F1519S in the plasma membrane was comparable to or higher than that of the WT, indicating that the hNav1.4 protein with F1519S was normally expressed in the plasma membrane with non-conducting ionic currents. Therefore, F1519S constitutes a variant that prevents ionic conduction through the primary ionic pore of hNav1.4.

Given that both L810Q and F1519S are loss-of-function variants, we concluded that hNav1.4 in the skeletal muscles of IV-1, IV-2, and IV-3 babies, was not sufficient to generate action potentials appropriately in skeletal msucle, resulting in fatal outcomes.

## Discussion

Hydrops fetalis is categorized into immune hydrops due to blood type (RhD)-incompatible pregnancy and NIHF. NIHF is estimated to occur in approximately one in 1700 to 3000 pregnancies, and associated with significant risks in stillbirth and neonatal death, and the risk of these adverse outcomes depends on the underlying cause of the edema [2, 23]. Appropriate administration of Rh(D) immunoglobulin results in approximately 90% hydrops fetalis cases being non-immune, with various genetic causes known to be linked to NIHF. The causes of fetal anomalies are often unknown, especially in cases of fetal death, and genetic diagnosis is seldom carried out because of ethical concerns. However, identifying the cause of fetal abnormalities can determine the child’s prognosis, and enhance understanding of the risk of recurrence, and provide valuable insights for subsequent fetal management and genetic counseling. In the present case, fetal death attributed to hydrops recurred thrice, prompting suspicion of an inheritable genetic cause because of its frequent recurrence. Consequently, we were prompted to search for genetic abnormalities. However, within the spectrum of prenatal abnormalities, many cases with phenotypic abnormalities are deemed non-abnormal through microarray and chromosome karyotyping, widely utilized in contemporary practice. In contrast, whole-exome sequencing (WES) proves capable of identifying pathologically relevant genetic variants in such cases [24]. In fact, conventional genetic testing methods, such as karyotyping and chromosomal microarray analysis, could only identify the cause in 25% of genetically tested NIHF cases [25]. Sparks et al. performed a WES analysis on 127 NIHF cases, and identified diagnostic genetic variants in 29% of the cases, with 68% of the total genetic variants being autosomal dominant and 27% being autosomal recessive [3]. A meta-analysis of 31 WES studies involving 445 NIHF cases reported an overall diagnostic rate of 37%. The predominant disease category observed was RASopathies, followed by neuromuscular diseases [26, 27]. Thus, in cases of NIHF where a genetic etiology is suspected but remains unresolved through standard testing, WES should be considered.

In our case, WES revealed a heterozygous missense variant, c.2429T>A, in *SCN4A* from the mother, and a heterozygous missense variant, c.4556T>C, in *SCN4A* from the father. Additionally, both *SCN4A* variants were identified in the three deceased children. As these variants were rare and not registered in ClinVar, their clinical significance remained undetermined. However, with functional analysis data, we reevaluated the pathogenicity of these variants, classifying both as likely pathogenic, with F1519S meeting the criteria PS3, PM1, PM2, and PP3 and L810Q satisfying the criteria PS3, PM2, and PP3.

Functional analysis in this study revealed that F1519S in Nav1.4, lacking ionic current, is a “null” variant, while L810Q is a partial loss-of-function variant. To date, several cases of compound heterozygous or homozygous *SCN4A* variants, exhibiting null and/or loss-of-function, have been reported in congenital myopathy, congenital myasthenic syndrome, or fetal dyskinesia [8, 10, 13, 14, 16]. In most cases, parents heterozygous for one of these variants did not present any neuromuscular symptoms, even in the case of the “null” variant. In addition, model mice with a null variant showed normal neuromuscular function, whereas homozygous null variants were lethal, suggesting that the *SCN4A* gene is well-tolerated for loss-of-function defects [28]. Although histological examinations of the skeletal muscle of the babies’ skeletal muscles were not available in this study, we speculated that both variants led to lethal consequences in NIHF owing to the impaired function of hNav1.4 expressed in the skeletal muscle.

Taken together, the clinical-genetic assessments and functional data of the mutant proteins unequivocally underscore the significance of *SCN4A* as a genetic cause of NIHF. Our study also highlights the clinical care value of extensive testing, including WES for NIHF, which can pave the way for preimplantation genetic testing, as in our case.
